# Repression of MUC1 Promotes Expansion and Suppressive Function of Myeloid-Derived Suppressor Cells in Pancreatic and Breast Cancer Murine Models

**DOI:** 10.3390/ijms22115587

**Published:** 2021-05-25

**Authors:** Mahnaz Sahraei, Mukulika Bose, J. Alexa Sanders, Chandrav De, Lopamudra DasRoy, Sritama Nath, Cory R. Brouwer, Pinku Mukherjee

**Affiliations:** 1 Department of Bioinformatics, Yale University, New Haven, CT 06520, USA; mahnaz@gmail.com; 2 Department of Biological Sciences, University of North Carolina at Charlotte, Charlotte, NC 28223, USA; cde@uncc.edu (C.D.); lopa@breastcancerhub.org (L.D.); pmukherj@uncc.edu (P.M.); 3 Department of Bioinformatics, University of North Carolina at Charlotte, Charlotte, NC 28223, USA; jsande50@uncc.edu (J.A.S.); cbrouwer@uncc.edu (C.R.B.); 4 Breast Cancer Hub, Concord, NC 28027, USA; 5 Wunderman Thompson Health IMsci, Cary, NC 27519, USA; sritama.nath@gmail.com

**Keywords:** pancreatic cancer, breast cancer, MDSCs, MUC1, immune suppression, iNOS, TGFβ, c–MYC, pSTAT3, Tregs

## Abstract

Myeloid-derived suppressor cells (MDSCs) are immature myeloid cells that are responsible for immunosuppression in tumor microenvironment. Here we report the impact of mucin 1 (MUC1), a transmembrane glycoprotein, on proliferation and functional activity of MDSCs. To determine the role of MUC1 in MDSC phenotype, we analyzed MDSCs derived from wild type (WT) and MUC1-knockout (MUC1KO) mice bearing syngeneic pancreatic (KCKO) or breast (C57MG) tumors. We observed enhanced tumor growth of pancreatic and breast tumors in the MUC1KO mice compared to the WT mice. Enhanced tumor growth in the MUC1KO mice was associated with increased numbers of suppressive MDSCs and T regulatory (Tregs) cells in the tumor microenvironment. Compared to the WT host, MUC1KO host showed higher levels of iNOS, ARG1, and TGF-β, thus promoting proliferation of MDSCs with an immature and immune suppressive phenotype. When co-cultured with effector T cells, MDSCs from MUC1KO mice led to higher repression of IL-2 and IFN-γ production by T cells as compared to MDSCs from WT mice. Lastly, MDSCs from MUC1KO mice showed higher levels of c-Myc and activated pSTAT3 as compared to MDSCs from WT mice, suggesting increased survival, proliferation, and prevention of maturation of MDSCs in the MUC1KO host. We report diminished T cell function in the KO versus WT mice. In summary, the data suggest that MUC1 may regulate signaling pathways that are critical to maintain the immunosuppressive properties of MDSCs.

## 1. Introduction

A diverse population of immature myeloid cells (IMCs) make up the myeloid-derived suppressor cells (MDSCs). This heterogenous population consists of cells (IMCs) with precursors for macrophages, granulocytes, or dendritic cells (DCs) that accumulate in chronic inflammation and tumor progression [1,2,3,4]. MDSCs are derived from the hematopoietic precursor cells in the bone marrow through modulation of myelopoiesis by inflammatory mediators [1,2,3,4,5]. MDSCs fail to differentiate into macrophages, granulocytes, and dendritic cells under cancer conditions [6,7]. In mice, MDSCs express both CD11b and Gr1 markers, and are mainly of two subtypes: polymorphonuclear Ly6G^+^Ly6C^lo^ (PMN) and monocytic Ly6G^–^Ly6C^hi^ (M) cells. In humans, these same subtypes can be characterized as Lin^-^HLA-DR^–^/^lo^CD33^+^ or Lin-HLA^–^DR^–^/^lo^CD11b^+^CD14^–^CD15^+^CD33^+^ for PMN-MDSCs and CD14^+^HLA^–^DR^neg^/^lo^ or Lin^-^HLA^–^DR^neg^/^lo^CD11b^+^CD14^+^CD15^-^ for M-MDSCs [1,2,5,8,9]. In murine tumor models, co-expression of the CD11b and myeloid cell lineage differentiation antigen GR1 is a distinctive MDSC phenotype marker [10]. MDSCs are reported as a major obstacle in achieving response to immune therapy because they exhibit highly immunosuppressive and tumorigenic activities [1,2,3,11,12]. MDSCs suppress anti-tumor immune response by diverse mechanisms, including deprivation of amino acids arginine and cysteine which are essential for T cell proliferation and anti-tumor immune response [1,13,14], by production of nitric oxide (NO) and reactive oxygen species (ROS) leading to nitration of T cell receptors (TCR) and by secretion of chemokines necessary for T cell migration and induction of apoptosis of T cells and NK cells [1,2,3,15,16]. MDSCs also produce interleukin-10 (IL-10) and transforming growth factor β 1 (TGFβ-1) that inhibit functions of immune effector cells [1,2,3,13,17], increase expression of programmed death-ligand 1 (PD-L1) [1,2,3,18] which can downregulate anti-tumor T cell-functions by interacting with PD-1 receptor expressed on T cells [19], secrete angiogenic factors like VEGF [20,21], and growth factors, matrix metalloproteinases, and cytokines promoting tumor growth and activation of regulatory T cells (Tregs) [2,22,23]. Therefore, MDSCs are important players in tumor-mediated immunosuppression.

MUC1 is a transmembrane mucin glycoprotein that is normally expressed in the glandular or luminal epithelial cells of the mammary gland, esophagus, stomach, duodenum, pancreas, uterus, prostate, and lungs, and in hematopoietic cells [24,25]. In normal cells, MUC1 is only expressed on the apical surface and is heavily glycosylated with the core protein sequestered by the carbohydrates. As cells transform to a malignant phenotype, expression of MUC1 increases several folds, and the expression is no longer restricted to the apical surface, but it is found all around the cell surface and in the cytoplasm [26]. In addition, glycosylation on tumor-associated MUC1 (tMUC1) is aberrant, with greater exposure of the peptide core than is found in normal tissues [27]. MUC1 was ranked as the second most optimal target out of 75 for immunotherapy by the National Cancer Institute [28]. Many studies have demonstrated a significant upregulation in the immunosuppressive functions of MDSCs after their migration to the tumor site. Inflammatory cytokines like interferon-γ (IFN-γ), interleukins 1, 4 and 13 (IL-1, IL-4, IL-13), tumor-necrosis factor-α (TNF-α), toll-like receptor (TLR) ligand, and prostaglandin E2 (PGE2) provide these activating signals that are mediated by STAT1, STAT6, NF-κB, and by increased activity of cyclooxygenase 2 (COX-2) [1,2,13,29,30,31,32]. COX2 has been shown to be regulated by mucin protein MUC1 in pancreatic cancer [33].

Poh et al. showed that development of bone marrow (BM) progenitors into CD11b^+^Gr1^+^ MDSCs is dependent on down-regulation of β–catenin levels, which is regulated by MUC1 [34]. We have shown previously that MUC1 expression in the tumor helps in maintaining the MDSCs in an immature and immunosuppressive state, leading to an aggressive nature of MUC1^+^ PDA tumors [35]. This makes tMUC1 on epithelial tumors an emerging target for immunotherapy [36,37]. Although MUC1 is known to be an epithelial cell marker, its expression and function on normal and neoplastic hematopoietic cells have been published [38,39]. Thus, the functional role of MUC1 in immature myeloid cells should be explored to further understand its regulatory mechanisms. In this study, we investigated the expansion, differentiation, and suppressive function of MDSCs in MUC1-wild type (WT) and MUC1-null (MUC1KO) mice under cancer conditions. We have used two models of epithelial tumors, namely, breast and pancreatic tumors since MUC1 is present in both types therefore making it important to show that targeting the normal MUC1 on MDSCs will lead to worse prognosis. In addition, recent studies have shown that the MDSC gene signature derived from a murine breast cancer model is highly translatable into human disease, thus indicating that the MDSC state is largely conserved between mice and human in breast cancer [40]. Also, many studies in pancreatic cancer models have shown the role of MDSCs in inhibiting the anti-tumor immune responses [41], thus showing the relevance of studying MDSCs in this tumor type.

All previous studies have reported the role of MUC1 expressed in the tumor microenvironment on MDSC recruitment and function. This is the first study that determines the role of normal MUC1 in the host organism on the expansion and immunosuppressive function of bone marrow derived MDSCs and downstream immune responses. Therefore, the tumor cells in this study are devoid of MUC1.

Using a pancreatic ductal adenocarcinoma (PDA) and a breast cancer cell line (both devoid of MUC1), we report that there is increased expansion of MDSCs in the MUC1KO mice, and that the MDSCs are highly suppressive as compared to that of MDSCs in WT mice. Since systemic targeting of MUC1 in several adenocarcinomas is being actively explored [42], the fact that MUC1 is involved in a regulatory mechanism of expansion and immunosuppressive activity of MDSCs is of high significance. From our data, it becomes clear that the therapeutic targeting of MUC1 must be specific to the tumor form of MUC1 because targeting normal MUC1 may lead to increased MDSCs.

## 2. Results

### 2.1. MUC1KO Mice Are More Susceptible to Tumor Growth

**Figure 1:**
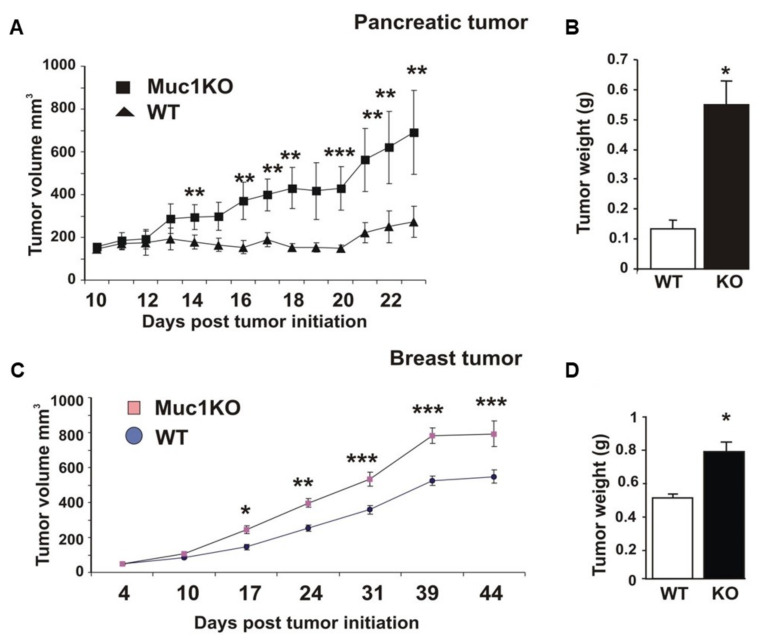
Increased tumor burden in MUC1KO mice. (**A**) For Pancreatic Cancer model, WT and MUC1KO mice were injected subcutaneously with 10^6^ cells and tumor burden was measured every 2 days. n = 8 mice in each WT and MUC1KO mice. (**B**) Final tumor weight in MUC1KO and WT mice injected with 10^6^ KCKO cells. (**C**) For Breast Cancer model, WT and MUC1KO mice were injected subcutaneously with 10^6^ cells and tumor burden was measured every 2 days. n = 6 and n = 7 mice in WT and MUC1KO groups respectively were used. (**D**) Final tumor weight in MUC1KO and WT mice injected with 10^6^ C57MG cells. Welch’s *t*-test was used to compare between WT and MUC1KO groups and *p* < 0.01 was considered significant. * *p* < 0.05, ** *p* < 0.01, *** *p* < 0.001.

MDSCs are an important component of tumor immunosuppressive microenvironment causing susceptibility to tumor growth [3]. In our study, a comparison of tumor growth and final wet weight between WT and MUC1KO mice indicate that in the absence of MUC1, mice are more susceptible to tumor growth with both KCKO and C57MG cell lines. In fact, not only tumors grew at a significantly faster rate in MUC1KO mice (Figure 1A,C), but the tumor weights were significantly higher in MUC1 KO mice compared to the WT mice (Figure 1B,D).

### 2.2. Increased Expansion and Migration of MDSCs to the Spleen of MUC1KO Mice and Higher Levels of TGF-β in Their Serum Compared to That of WT Mice

**Figure 2:**
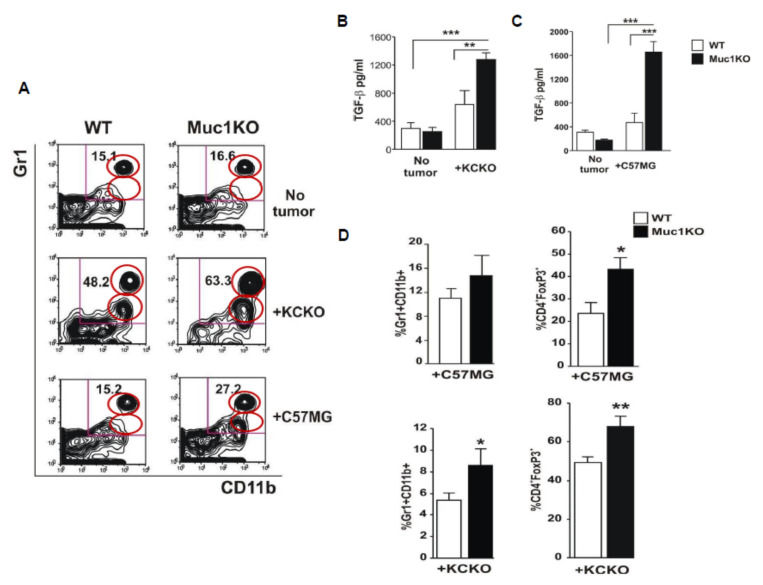
Cancer bearing MUC1KO mice have higher frequency of MDSCs creating a more immune suppressive environment. (**A**) Splenocytes from healthy and tumor bearing WT and Muc1KO mice were isolated and labeled with anti Gr1-APC and CD11b-PECy7 antibodies. Representative contour plots of Gr1^+^CD11b^+^ cells from three separate experiments are shown; the MDSC subsets were obtained by gating on high SSC and high FSC cells using an isotype control antibody and further on high Gr1^+^ and low Gr1^+^ CD11b^+^ cells. The red circles represent the percent depicted on the right top quadrant for each of the groups. (**B**,**C**) Levels of TGF-β was measured in the serum of healthy and cancer bearing WT and MUC1KO mice using ELISA kits. (**D**) Levels of MDSCs and CD4^+^FoxP3^+^ cells were measured in the tumor of WT and MUC1KO mice using flow cytometry. High SSC and FSC was gated for the lymphocyte population, then CD4^+^ T cells were gated on the lymphocyte population, and the CD25^+^ FoxP3^+^ double positive cells were gated on the CD4^+^ T cell population. Welch’s *t*-test was used to compare between WT and MUC1KO groups and *p* < 0.01 was considered significant. * *p* < 0.05, ** *p* < 0.01, *** *p* < 0.001.

Spleen is the first major lymphatic organ to which MDSCs migrate to exert their suppressive function prior to infiltrating tumors [1,2,3]. To study whether MUC1 regulates expansion and migration of MDSCs to the spleen of tumor bearing mice, we analyzed splenocytes from these mice for Gr1^+^CD11b^+^ MDSC level by flow cytometry. Both breast cancer C57MG and pancreatic cancer KCKO cells induced significant expansion/migration of MDSCs into the spleen, and MDSC expansion was significantly higher in the MUC1KO mice compared to the WT mice in the spleen and the tumor (Figure 2A,D). Due to the increased expansion of MDSCs in MUC1KO mice, we wanted to compare the serum concentration of TGF-β between the WT and MUC1KO mice. It has been demonstrated that TGF-β induces MDSC expansion [43,44]. Our results show that in both cancer models, levels of TGF-β were significantly higher in MUC1KO versus the WT mice (Figure 2B,C). Increased suppressive activity of MDSCs is associated with increased level of CD4^+^/CD25^+^/FoxP3^+^ Treg cells in the tumor environment [6]. Thus, we hypothesized that lack of MUC1 and its association with increased MDSCs enhances the numbers of Tregs in the tumor. Indeed, we found that lymphocytes derived from KCKO and C57MG tumors in MUC1KO mice had higher numbers of CD4^+^/CD25^+^/FoxP3^+^ Treg cells and MDSCs than those derived from WT mice (Figure 2D). Overall, our results demonstrate that during cancer development, MUC1KO mice have higher levels of the immunosuppressive cytokine TGF-β and increased numbers of MDSCs and Tregs.

### 2.3. Spleen-Derived MDSCs from MUC1KO Mice Have Higher Immunosuppressive Phenotype

**Figure 3:**
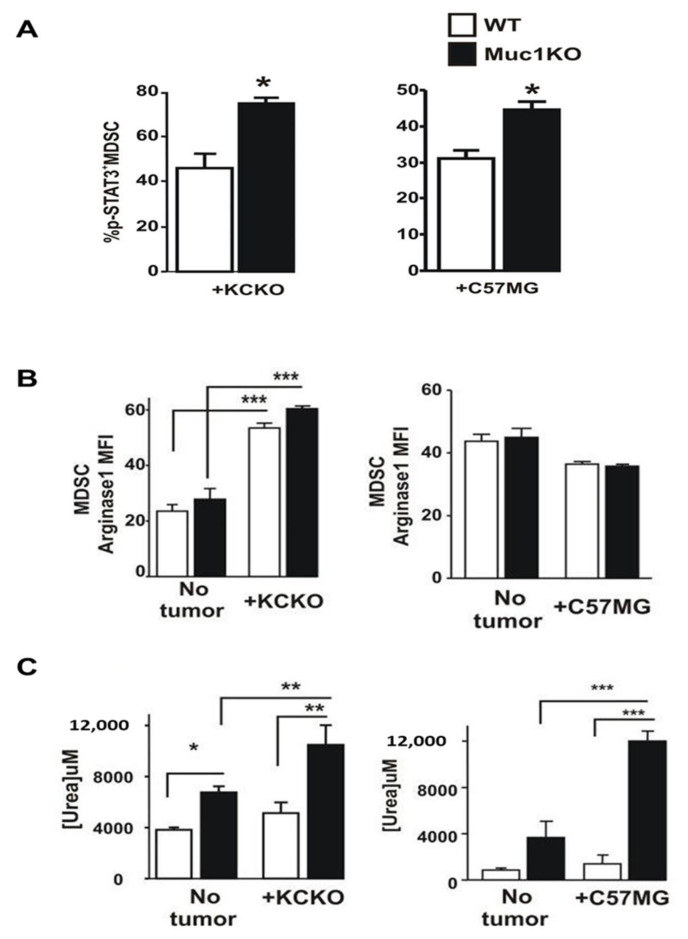
Phenotypic characterization of MDSCs in the BM of healthy and tumor bearing WT and Muc1KO mice. (**A**) Splenocytes from tumor bearing WT and MUC1KO mice were isolated and labeled with antibodies against Gr1, CD11b, and pSTAT3. pSTAT3^+^ cells were determined on Gr1^+^ CD11b^+^ gated cells. (**B**) MFI values of Arginase-1 expression in MDSCs of WT and MUC1KO mice was measured via flow cytometry. Arg-1^+^ cells were determined on Gr1^+^ CD11b^+^ BM gated cells. (**C**) Arginase-1 activity on sorted MDSCs was measured using a urea assay. Welch’s *t*-test was used to compare between WT and MUC1KO groups and *p* < 0.01 was considered significant. * *p* < 0.05, ** *p* < 0.01, *** *p* < 0.001.

Since phosphorylated STAT3 (pSTAT3) is an enhancer of stemness and mesenchymal properties of MDSCs [45,46], we assessed pSTAT3, ARG1, and urea levels in splenic derived MDSCs from MUC1KO versus WT mice. We observed an increased level of pSTAT3 in MDSCs derived from tumor-bearing MUC1KO versus WT mice (Figure 3A). Activated STAT3 pathway is known to aid expansion of MDSCs and prevent maturation of myeloid progenitor cells [46]. When we assessed the ARG1 levels using flow cytometry, to our surprise, we observed that MDSCs from KCKO tumor-bearing MUC1KO versus WT mice showed similar increases in ARG1 compared to their non-tumor bearing counterparts (Figure 3B), whereas no such increase in ARG1 levels was noted in the C57MG tumor-bearing mice (Figure 3B). Thus, we further determined if differences existed in the ARG1 activity. Since ARG1 converts L-arginine to urea [47], we tested the levels of urea production in Gr1^+^CD11b^+^ MDSCs sorted from the spleen of tumor-bearing WT/KO mice. Interestingly, we found that the urea production in MDSCs from MUC1KO mice was significantly higher than that of its WT counterparts indicating a more suppressive phenotype of MDSCs in MUC1KO mice (Figure 3C).

### 2.4. MDSCs from the Spleen of MUC1KO Tumor Bearing Mice Lead to Increased Suppression of Cytotoxic T Cells Compared to WT MDSCs

**Figure 4:**
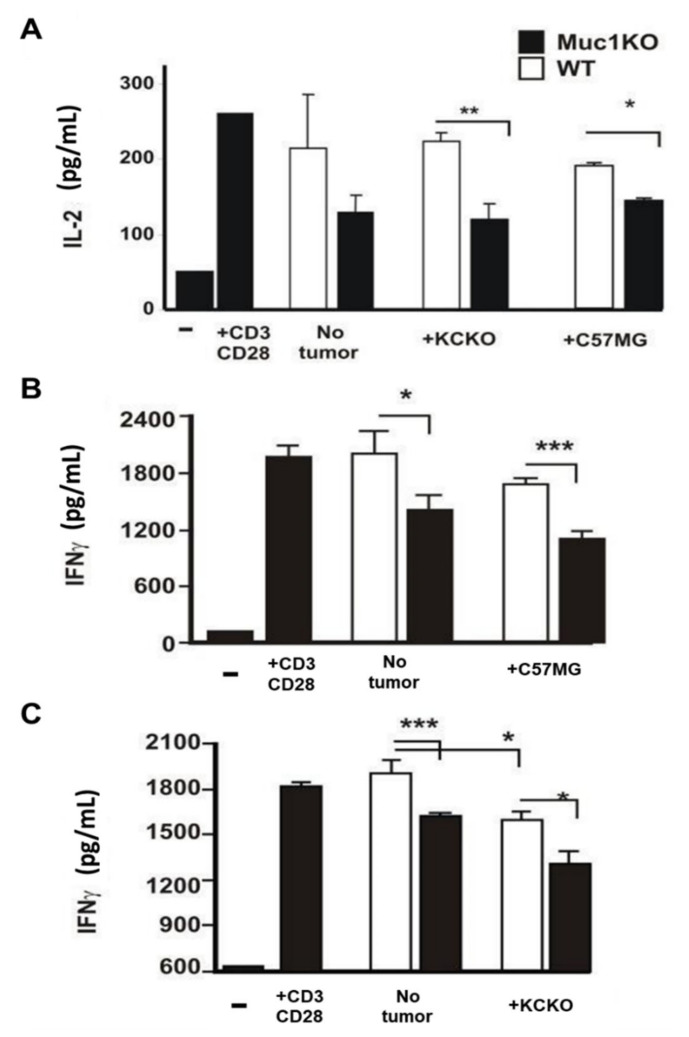
MDSCs from MUC1KO mice inhibit production of proinflammatory cytokines IL-2 and IFN-γ by T cells. (**A**) MDSCs were sorted from the spleen of WT and MUC1KO mice and incubated with activated syngeneic T cells. IL-2 secretion was measured via ELISA. (**B**,**C**) Analysis of IFN-γ secretion from CD3/CD28 activated T cells incubated with WT and MUC1KO MDSCs from healthy and tumor bearing mice. Welch’s *t*-test was used to compare between WT and MUC1KO groups and *p* < 0.01 was considered significant. * *p* < 0.05, ** *p* < 0.01, *** *p* < 0.001.

Splenic derived MDSCs from WT and MUC1KO mice bearing C57MG and KCKO tumors were co-cultured with naïve syngeneic T cells stimulated with α-CD3/CD28 antibodies and supernatants were collected to test for IL-2 and IFN-γ production. Our results showed that MDSCs from the spleen of tumor bearing MUC1KO mice were more effective in inducing T cell suppression indicated by lower IL-2 (Figure 4A) and IFN-γ production by T cells (Figure 4B,C). It must be noted that MDSCs derived from non-tumor bearing MUC1KO but not WT mice induced suppression of IL2 and IFN-γ production by T cells. These data indicate that MUC1 plays an important role in inducing and regulating the suppressive function of MDSCs. It is also recently published that MUC1 is a critical driver of MDSC expansion in patients with AML [48].

In addition, we conducted flow cytometry to show T cell phenotype and function from the splenocytes and peripheral blood leukocytes (PBLs) from MUC1KO versus WT mice. Supplementary Figure S1 shows the altered phenotype of the T cells suggesting impaired activation of T cells in KO versus WT mice. Although there was no significant difference in the percentages of CD4 and CD8 T cells in the spleen or PBLs (a and b), the expression of T cell signaling molecules including CD3ε, TCRαβ, and CD3ζ were significantly lower in T cells from KO versus the WT mice (c and d). Furthermore, T cells from WT and MUC1KO mice differed significantly in the levels of CD69 and IL2R levels (e and f) in response to α-CD3ε+α-CD28 stimulation suggesting impaired activation via TCR. In Supplementary Figure S2, we show that T cells from MUC1KO mice had significantly lower proliferation as compared to T cells from WT mice in response to (a) ConA, (b) α-CD3ε+α-CD28, or (c) in vivo Ovalbumin (Ova) stimuli. Proliferation of T cells in response to ConA and α-CD3ε+α-CD28 was in fact partially restored when MUC1 gene was added back in the KO mice for T cell. (MUC1.Tg mice have the human MUC1 and the mouse Muc1 and were used as control. This mouse model has been well published since 1998 [49])

### 2.5. BM-MDSCS from MUC1KO Mice Have Different Rates of Expansion and Proliferation Compared to That from WT Mice

**Figure 5:**
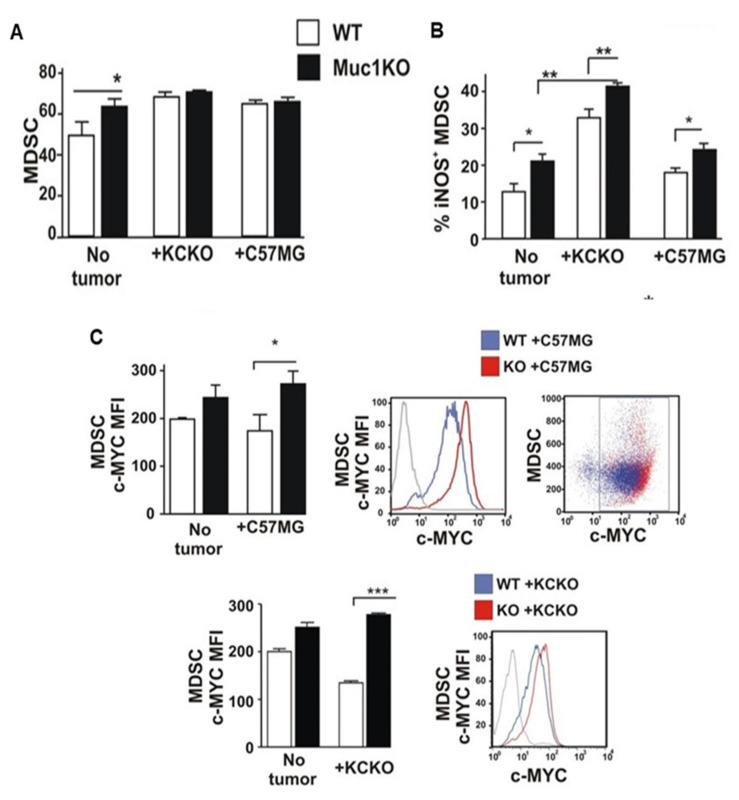
MDSCs from MUC1KO mice produce increased iNOS and c-Myc. (**A**) BM cells from healthy mice were isolated and labeled with antibodies against Gr1 and CD11b. (**B**) Level of iNOS expression by MDSCs from the BM of healthy and tumor bearing mice. Percentages of iNOS^+^ cells on Gr1^+^ CD11b^+^ BM gated cells. (**C**) MFI values of c-Myc protein expression by MDSCs from the BM of healthy and tumor bearing mice. c-Myc^+^ cells were gated in Gr1 CD11b^+^ cells. Welch’s *t*-test was used to compare between WT and MUC1KO groups and *p* < 0.01 was considered significant. * *p* < 0.05, ** *p* < 0.01, *** *p* < 0.001.

To study whether MUC1 expression on hematopoietic stem cells (HSCs) leads to differential expansion of MDSCs in the bone marrow (BM) of non-tumor bearing mice, freshly isolated BM cells were analyzed for co-expression of Gr1 and CD11b. We found that MUC1KO mice have 15% higher levels of MDSCs in the BM indicating that MUC1 may regulate ontogeny of these cells (Figure 5A).

To characterize the functional activity of BM-MDSCs from WT and MUC1KO mice, we studied levels of iNOS expression by flow cytometry. Our results indicate that higher percentage of MDSCs from healthy non-tumor-bearing MUC1KO mice express iNOS (Figure 5B) than its WT counterparts. Moreover, MDSCs from both breast C57MG and pancreatic KCKO tumor-bearing MUC1KO mice express higher levels of iNOS than MDSCs derived from WT mice, suggesting a more suppressive phenotype in MUC1KO MDSCs compared to WT MDSCs (Figure 5B).

To check whether MDSCs from WT and MUC1KO mice differ in their survival and proliferation capabilities, we checked the levels of c-Myc protein. c-Myc is shown to be responsible for survival and proliferation of MDSCs [50]. Furthermore, MUC1 induces increased expression of c-Myc that induces proliferation in the MDSC population via downstream effects on cell cycle proteins [48]. We determined levels of c-Myc expression on MDSCs and found that under tumor condition, BM-MDSCs from MUC1KO mice expresses significantly higher levels of c-Myc than the WT MDSCs (Figure 5C). Our data suggest that in the absence of MUC1, BM-MDSCs are more likely to survive, proliferate, and probably have a longer life span. This may explain the data in Figure 1 where there is enhanced tumor growth in MUC1KO versus WT mice.

## 3. Discussion

Previously, we have published the differences in the MDSC function in a MUC1-positive versus negative tumor and the downstream effects on anti-tumor immunity [35]. However, in this study, we focused on MUC1 in the host (but not in the tumor). We therefore used the MUC1 KO and WT mice and selected MUC1-negative tumors. The novelty of this study is the focus on the changes in MDSC phenotype and function in genetically identical host systems in which the only MUC1 gene is either present or absent. We further report alterations in downstream immune signaling and reduced T cell activation and function in the MUC1 WT versus KO host environment.

Gr1^+^CD11b^+^ MDSC expansion appears in pathologic conditions of chronic inflammation including cancer [10]. It is critical to understand the mechanisms that regulate the functional activity of MDSCs which play a significant role in tumor angiogenesis, drug and immunotherapy resistance, and promotion of tumor metastases. The two groups of signaling previously described to be responsible for accumulation and differentiation of MDSCs include tumor—derived growth factors (STAT3, IRF8, C/EBPβ, Notch, adenosine receptors A2b signaling, NLRP3, Rb1) and proinflammatory cytokines produced by tumor stroma (NF-κβ pathway, STAT1, STAT6, PGE2, COX2) [7]. At the same time, there were fewer reports regarding the downstream targets of tumor derived factors such as MUC1 that contains binding sites for NF-κβ in its promoter and that depends on STAT factors for its expression (reviewed in [42,51]). The latter finding defines that development of BM progenitors into MDSCs is mediated through down-regulation of β-catenin levels in the absence of MUC1 in vitro [34]. Here, we report that MUC1 regulates proliferation and suppressive phenotype of MDSCs in both healthy and cancer conditions. Using two different tumor models, we were able to show that, in MUC1KO host, the MDSC numbers and function is increased as compared to its WT counterpart with intact MUC1.

Previous studies showed that MDSCs influence differentiation of CD4^+^ cells to Treg cells through the production of TGF-β cytokine [52,53]. Preliminarily, proteomics analysis conducted on MUC1-positive and MUC1-negative tumors show differential expression of immune pathways and protein-protein network (Supplementary Figure S3). The proteomics data show how tumor-associated MUC1 can influence the tumor microenvironment by mechanisms such as (1) upregulating or downregulating multiple targets (2) which lead to amino acid depletion, maintenance of a hypoxic environment, and ROS production, (3) thus leading to reduced anti-tumor activity of the lymphocytes. Targeting the tumor MUC1 is therefore an essential mechanism to increase immunogenicity of the tumor.

The knockout efficiency in the MUC1KO mouse model is shown by immunohistochemistry (Supplementary Figure S4). We found that absence of MUC1 in the MUC1KO host was associated with higher expansion of MDSCs and more immunosuppressive phenotype. These effects may be mediated through the increased levels of pSTAT3 and c–Myc that are responsible for the survival, immature phenotype, and immunosuppressive features of MDSCs [7,46,54].

Future research will be focused on understanding the mechanisms of MUC1-regulated expression of c-Myc in MDSCs. It was reported that MUC1 may regulate c-Myc expression on transcriptional level [55,56]. Alternative mechanisms of MUC1 regulation might include STAT3-mediated overexpression of c-Myc and the downstream effects on cell cycle proteins [48,57]. We report increased levels of pSTAT3 in the spleen-derived MDSCs from MUC1KO tumor-bearing mice compared to those from WT tumor-bearing mice. MUC1-mediated increased activity of pSTAT3 deregulates expression of c-Myc and may be a reason behind the observed overexpression of c-Myc in MDSCs.

**Figure 6:**
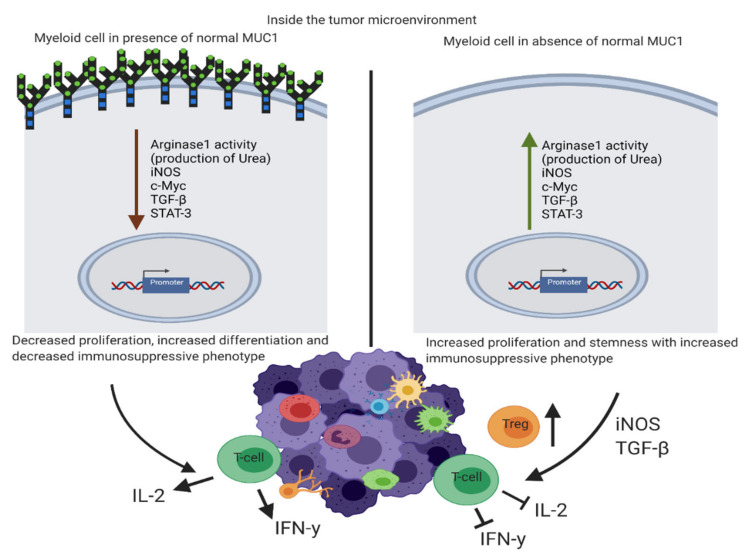
Schematic diagram showing the differences in the tumor microenvironment of a MUC1KO mouse versus that of a WT mouse. Schematic diagram showing the differences in the tumor microenvironment of a MUC1KO mouse (right) vs that of a WT mouse (left). Red arrow indicates downregulation and green arrow indicates upregulation of genes. MUC1KO mouse have increased TGF-β and T-reg cells in their tumor microenvironment thus. MDSCs from MUC1KO mouse produce higher urea, iNOS, stemness marker Sca1, and TGF-β, and show increased expansion and migration into the tumor, thus inhibiting T cells from producing IL-2 and IFN-γ.

We have used the MUC1-negative tumor cells so that we can focus on the role of host MUC1 (Supplementary Figure S4 shows that the tumors have no MUC1 staining by IHC). We report the critical role of host normal MUC1 in regulating the suppressive phenotype of MDSCs as summarized in Figure 6. Lack of MUC1 correlated with increased levels of iNOS, ARG1 activity, and TGF-β secretion that are markers of MDSC’s immunosuppressive activity [58]. It is reported that iNOS induces NO leading to inhibition of T cells via downregulation of IL-2 production and receptor signaling and induction of T cell apoptosis [59]. ARG1—dependent depletion of L-arginine impairs T cell proliferation and function [12,52,59]. We were able to demonstrate a significant impact of MUC1-mediated increased iNOS and ARG1 activity on T cell function including repression of IL-2 and IFN-γ production. Thus, knockdown of MUC1 in MDSCs may have a negative effect on the immune responses against the tumor [4].

Previously, the paradoxical roles of MUC1 based on its pattern of glycosylation in normal versus cancer cells have been reported in case of cancer-associated infections [60]. We have shown that there is significant difference in immune response in MUC1^+^ versus MUC1 deficient tumors [35]. We demonstrate how immune pathways are differentially regulated in MUC1^+^ versus negative tumors (Supplementary Figure S3). The data show that, in future, specific targeting of tumor-associated MUC1 (tMUC1) without targeting the normal MUC1 on immune cells should be a preferred strategy to increase efficacy against tumors. While we report that normal MUC1 on the MDSC populations reduce their expansion, proliferation, migration to the spleen, and immunosuppressive activities, this should not be confused with the tumor-associated MUC1 (tMUC1) which when expressed on tumor cells increases the aggressive phenotype of the tumor and is a marker for poor prognosis. Data clearly demonstrates that elimination of MUC1 in the host leads to the generation of immature immunosuppressive MDSCs which potentiates tumor growth. Therefore, any MUC1 targeted therapy must take into account not to disturb the normal MUC1, and thus a recently reported novel monoclonal antibody TAB004 (OncoTAb, Inc., Charlotte, NC, USA), which specifically targets only the tumor-associated form of MUC1 (tMUC1) may serve as the perfect delivery agent for targeting tumor MUC1 while sparing normal MUC1 [36,61,62].

## 4. Materials and Methods

### 4.1. Cell Lines

PDA cell line KCKO was generated from spontaneous PDA MUC1-null tumors and maintained as previously described [63]. C57MG breast cancer cells were kindly provided by Dr. Gendler, Mayo clinic, and maintained according to the published protocol [64].

### 4.2. Mouse Models

C57BL/6 mice were obtained from The Jackson Laboratory and MUC1KO mice were generated as previously described [65]. Mice were handled and maintained in accordance with the University of North Carolina at Charlotte Institutional Animal Care and Use Committee approved protocol. Mice were 3 months old at the time of tumor injection. For the PDA model, 10^6^ KCKO cells were injected in the right flank of the male mice. For the breast cancer model, 10^6^ C57MG cells were injected in the right lower mammary fat pad of female C57BL/6 mice. Palpable tumors were measured by calipers every two days, and tumor weight was calculated according to the following formula: milligrams = (length × (width)^2^)/2, where length and width are measured in centimeters.

### 4.3. Flow Cytometry

A total of 5 × 10^5^ bone marrow cells were obtained from the femurs and tibias of mice. Splenocytes from control and tumor bearing mice were derived as previously described [35]. Tumor infiltrating lymphocytes were isolated by digesting tumors with 0.5 mg/mL Collagenase III (Worthingthon Biotech, NJ) for 20 min at 37 °C. Tumors were incubated with DNAse I for 10 min at room temperature. Samples were passed through a 40 uM screen and finally centrifuged in 40/60 Percoll for 20 min. Lymphocytes were collected and then stained with fluorescence-conjugated antibodies. Cells were stained with anti-Gr1-APC, anti CD11b-PE, anti-Ly6C-FITC, anti-Ly6G-APC, anti-CD11c-FITC, and biotinylated anti-CD115 for total MDSC population, subsets, and maturation phenotypes, respectively. For the detection of intracellular antigens, the cells were fixed and simultaneously permeabilized with BD Cytofix/Cytoperm and stained with anti iNOS-FITC and anti-Arg-1 primary antibody and appropriate secondary APC conjugated antibody. Arg-1^+^ cells were gated on Gr1^+^ CD11b^+^ BM gated cells. Splenocytes were harvested from spleens of 6- to 8-week-old C57BL/6 mice under sterile conditions, red blood cells were lysed (1 × RBC lysing buffer), and single-cell suspensions were prepared for staining with anti-Gr1-APC and anti-CD11b-PE antibodies. Single-cell suspensions were prepared for staining with anti-Gr1-APC and anti-CD11b-PE antibodies. Samples were run on a BD FACS Calibur and BD Fortessa (BD Biosciences, San Jose, CA, USA) and data analyzed using the FlowJo software (Tree Star Inc., Ashland, OR, USA). Data are expressed as percentage positive cells and/or mean fluorescence intensity (MFI). The MDSC subsets were obtained by gating on high SSC and high FSC cells using an isotype control antibody and further on high Gr1^+^ and low Gr1^+^ CD11b^+^. For Tregs, cells were stained with CD4, CD25 and FoxP3 antibodies. High SSC and FSC was gated for the lymphocyte population, then CD4^+^ T cells were gated on the lymphocyte population, and the CD25^+^ FoxP3^+^ double positive cells were gated on the CD4^+^ T cell population.

### 4.4. Antibodies

Fluorescent antibodies against Gr1 (Miltenyi Biotech), CD11b (BD), iNOS (BD), Arginase-1 (BD), c-Myc (SantaCruz Biotech), MUC1 (BD), pSTAT3 (eBioscience), CD4, CD8, CD25, FoxP3, CD3, TCR, CD69, IL-2R (BD) and isotype control (eBioscience) were used at a concentration of 1 µg/sample.

### 4.5. Arginase Assay

A total of 2 × 10^5^ MDSCs were subjected to Arginase activity assay as described before [66,67]. Briefly, cells were lysed with 1% Triton-X in 37 °C. 25 mM Tris-HCl and 10 mM MnCl_2_ were added to samples. Lysates were then heated at 56 °C to activate Arginase-1. 0.5 M L-arginine was added to the lysates and the reaction was stopped using phosphoric and sulfuric acid. 40 µL of a-isonitrosopropiophenone was added and samples were heated at 95 °C for 30 min. Samples were read at 450 nm.

### 4.6. ELISA

Levels of cytokine TGF-β in the murine serum was analyzed with a kit from eBioscience (cat. no. 88-8350-22) using sandwich ELISA.

### 4.7. MDSC Suppression Assay

CD3^+^ T cells were isolated from healthy C57/B6 mice using a Miltenyi kit (Miltenyi Biotec, Auburn, CA cat. no 130-050-101). Round bottom plates were coated with 10 µg of functional CD3 antibody (eBiosciences, San Diego, CA, USA) and 5 µg CD28 antibody overnight. A total of 10^5^ CD3^+^ T cells were plated in RPMI containing 10% heat inactivated fetal calf serum, 1% penicillin/streptomycin and 1% glutamax (Invitrogen). MDSCs were sorted first by enriching the CD11b^+^ cells from the spleen and then sorted for Gr1^+^cells using a Miltenyi kit cat. no. 130-049-601. As MDSCs populations can be sorted by presence of Gr1 marker alone and are known to possess suppressive activity on day 5 [68], Gr1^+^cells were sorted using anti-APC microbeads (Miltenyi, Bergisch Gladbach, Germany) and anti-Gr1-APC antibody (BD Biosciences, San Jose, CA, USA). The purity of the sorted cell fraction was >90%. A total of 10^5^ MDSCs were incubated with CD3^+^ T cells for 72 h. Supernatants were collected at 72 h and were then subjected to IL-2 and IFN-γ ELISA assays (eBiosciences cat. nos. BMS601TEN and BMS606-2).

### 4.8. T Cell Proliferation Assay

Cells were cultured for 24 h at 37 °C in a 10% CO_2_ incubator and 1 µCi of 3H-thymidine was added to each well 16 h prior to harvesting. Data are presented as 3H-thymidine incorporation in cpms. Proliferation was determined by measuring 3H-thymidine incorporation using the beta plate counter (Packard Instruments, Perkin Elmer Life Sciences, Boston, MA, USA) as described previously [69]. In some experiments, Concanavalin A (Sigma, St. Louis, MO, USA), CD3+CD28 antibodies (Invitrogen, Carlsbad, CA, USA) and Ovalbumin (Sigma, St. Louis, MO, USA) were added to the co-culture at increasing concentrations. Isotype control antibodies at the same concentration were used as negative controls in these assays.

### 4.9. Immunohistochemistry

For nonenzymatic antigen retrieval, sections were heated to 85 °C in Dako antigen retrieval solution for 90 min and cooled for 20 min; all subsequent steps occurred at room temperature. To quench endogenous peroxidase, slides were rinsed and incubated in methanol/2% H_2_O_2_ for 10 min. Sections were then washed, blocked in 50% fetal bovine serum (FBS) in PBS for 45 min, and incubated overnight with primary antibodies. Sections were incubated for 1 h with secondary antibody, developed with a diaminobenzidine (DAB) substrate (Vector Inc., Burlingame, CA, USA), counterstained with hematoxylin, and mounted with Permount. Primary antibody used was Armenian hamster anti-MUC1 cytoplasmic tail (CT), CT2 (1:50) and secondary antibody used was rat anti-hamster (1:100, Jackson Labs) IgG conjugated to horseradish peroxidase. Immunopositivity was assessed using light microscopy and images taken at 100× or 200× magnification.

### 4.10. Proteomics

Proteomics data collected from a previous analysis [70] in which protein spectral counts were analyzed from PDA.MUC1 and PDA.MUC1 knock out mice was used for further analysis. Fold change was calculated between MUC1 and KO. Proteins with a 2-fold difference between the two samples were considered differentially expressed. Genes associated with these differentially expressed proteins were input into Ingenuity Pathway Analysis (IPA) to identify any associated pathways that are related to cellular immune response (QIAGEN Inc., Germantown, MD, USA, https://www.qiagenbioinformatics.com/products/ingenuity-pathway-analysis, accessed on 15 March 2021). Pathways with a *p*-value of less than 0.05 were considered significant and are shown in Supplementary Figure S3.

### 4.11. Statistical Analysis

Statistical analysis was performed with GraphPad Prism 6.0 software, San Diego, CA, USA. One-way ANOVA was performed between the groups and the significance was confirmed using the Duncan and Student-Newman-Keul test. Values were considered significant if *p* < 0.05.

## Supplementary Materials

Supplementary materials can be found at https://www.mdpi.com/article/10.3390/ijms22115587/s1.
