# Supplementary material for: Repression of MUC1 Promotes Expansion and Suppressive Function of Myeloid-Derived Suppressor Cells in Pancreatic and Breast Cancer Murine Models
Source: Int J Mol Sci. 2021 May 25;22(11):5587. doi: 10.3390/ijms22115587 (PMC8197523; doi:10.3390/ijms22115587)
Supplement: Supplementary file 1 [file ijms-22-05587-s001.zip › Supplemental figures.pptx]

## Slide 1
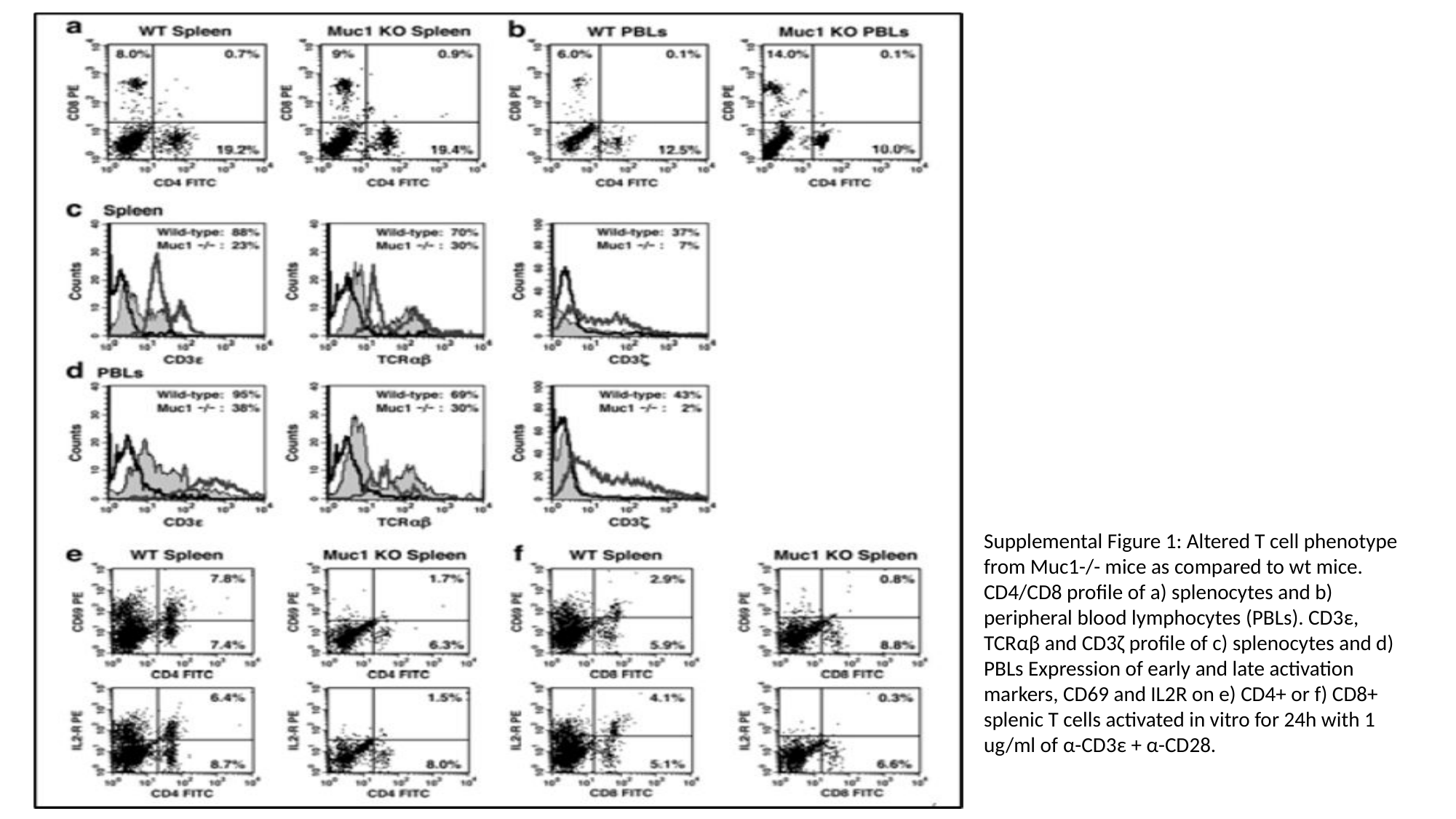

Supplemental Figure 1: Altered T cell phenotype from Muc1-/- mice as compared to wt mice. CD4/CD8 profile of a) splenocytes and b) peripheral blood lymphocytes (PBLs). CD3ε, TCRαβ and CD3ζ profile of c) splenocytes and d) PBLs Expression of early and late activation markers, CD69 and IL2R on e) CD4+ or f) CD8+ splenic T cells activated in vitro for 24h with 1 ug/ml of α-CD3ε + α-CD28.

## Slide 2
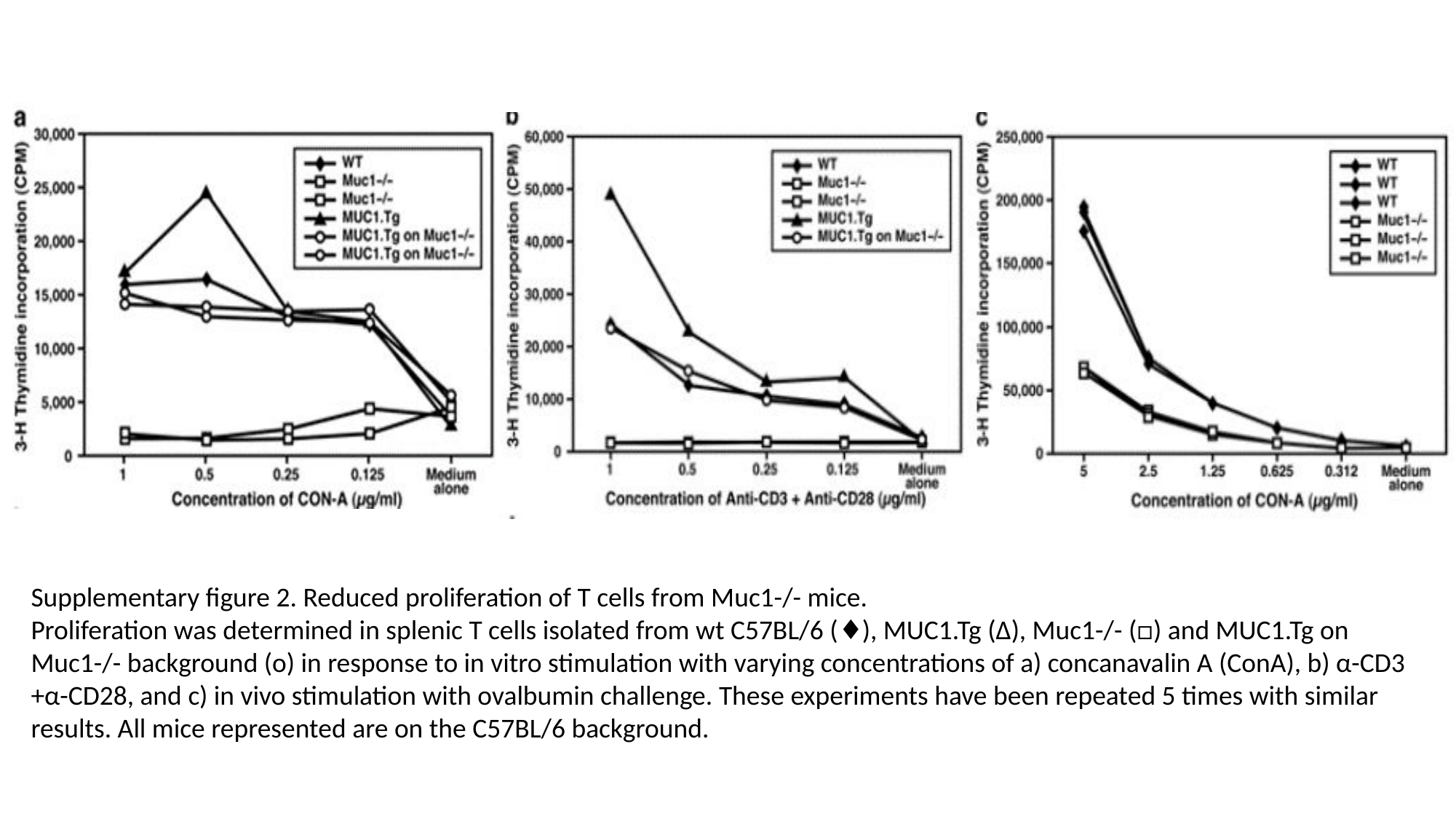

#
Supplementary figure 2. Reduced proliferation of T cells from Muc1-/- mice.
Proliferation was determined in splenic T cells isolated from wt C57BL/6 (♦), MUC1.Tg (Δ), Muc1-/- (□) and MUC1.Tg on Muc1-/- background (ο) in response to in vitro stimulation with varying concentrations of a) concanavalin A (ConA), b) α-CD3 +α-CD28, and c) in vivo stimulation with ovalbumin challenge. These experiments have been repeated 5 times with similar results. All mice represented are on the C57BL/6 background.

## Slide 3
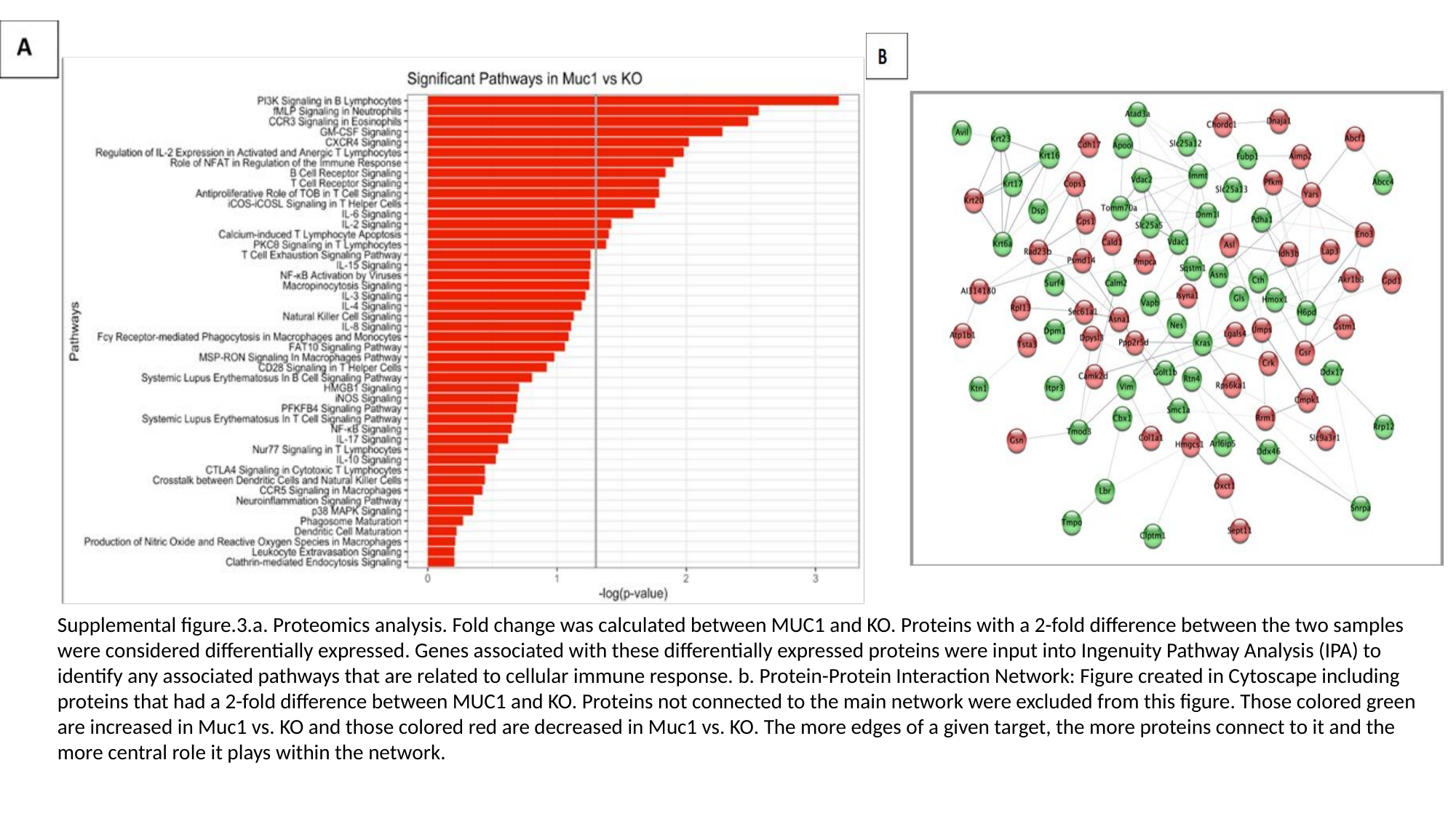

#
Supplemental figure.3.a. Proteomics analysis. Fold change was calculated between MUC1 and KO. Proteins with a 2-fold difference between the two samples were considered differentially expressed. Genes associated with these differentially expressed proteins were input into Ingenuity Pathway Analysis (IPA) to identify any associated pathways that are related to cellular immune response. b. Protein-Protein Interaction Network: Figure created in Cytoscape including proteins that had a 2-fold difference between MUC1 and KO. Proteins not connected to the main network were excluded from this figure. Those colored green are increased in Muc1 vs. KO and those colored red are decreased in Muc1 vs. KO. The more edges of a given target, the more proteins connect to it and the more central role it plays within the network.

## Slide 4
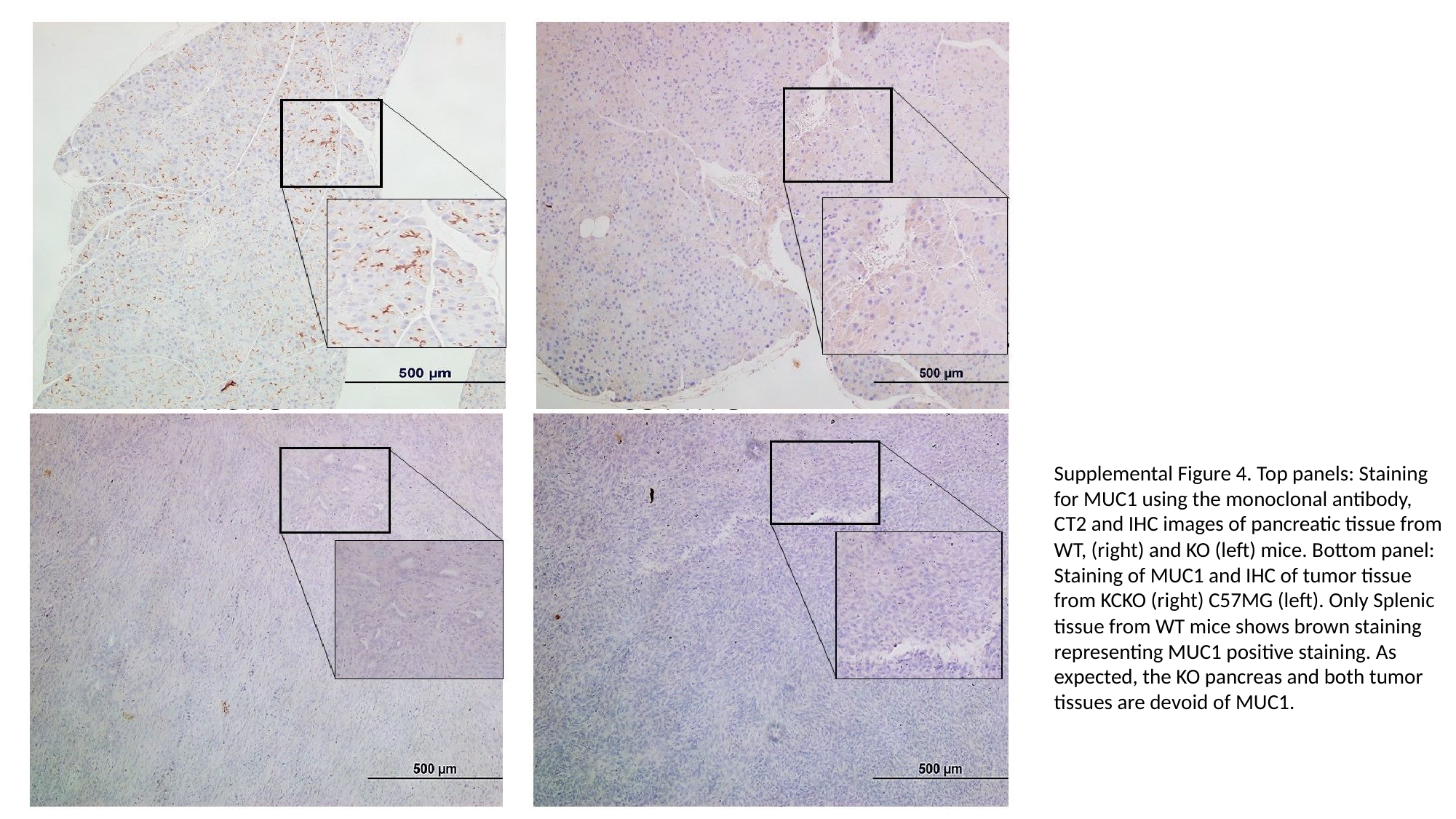

#
Supplemental Figure 4. Top panels: Staining for MUC1 using the monoclonal antibody, CT2 and IHC images of pancreatic tissue from WT, (right) and KO (left) mice. Bottom panel: Staining of MUC1 and IHC of tumor tissue from KCKO (right) C57MG (left). Only Splenic tissue from WT mice shows brown staining representing MUC1 positive staining. As expected, the KO pancreas and both tumor tissues are devoid of MUC1.
