# Supplementary figures and images for: Repression of MUC1 Promotes Expansion and Suppressive Function of Myeloid-Derived Suppressor Cells in Pancreatic and Breast Cancer Murine Models
Source: Int J Mol Sci. 2021 May 25;22(11):5587. doi: 10.3390/ijms22115587 (PMC8197523; doi:10.3390/ijms22115587)

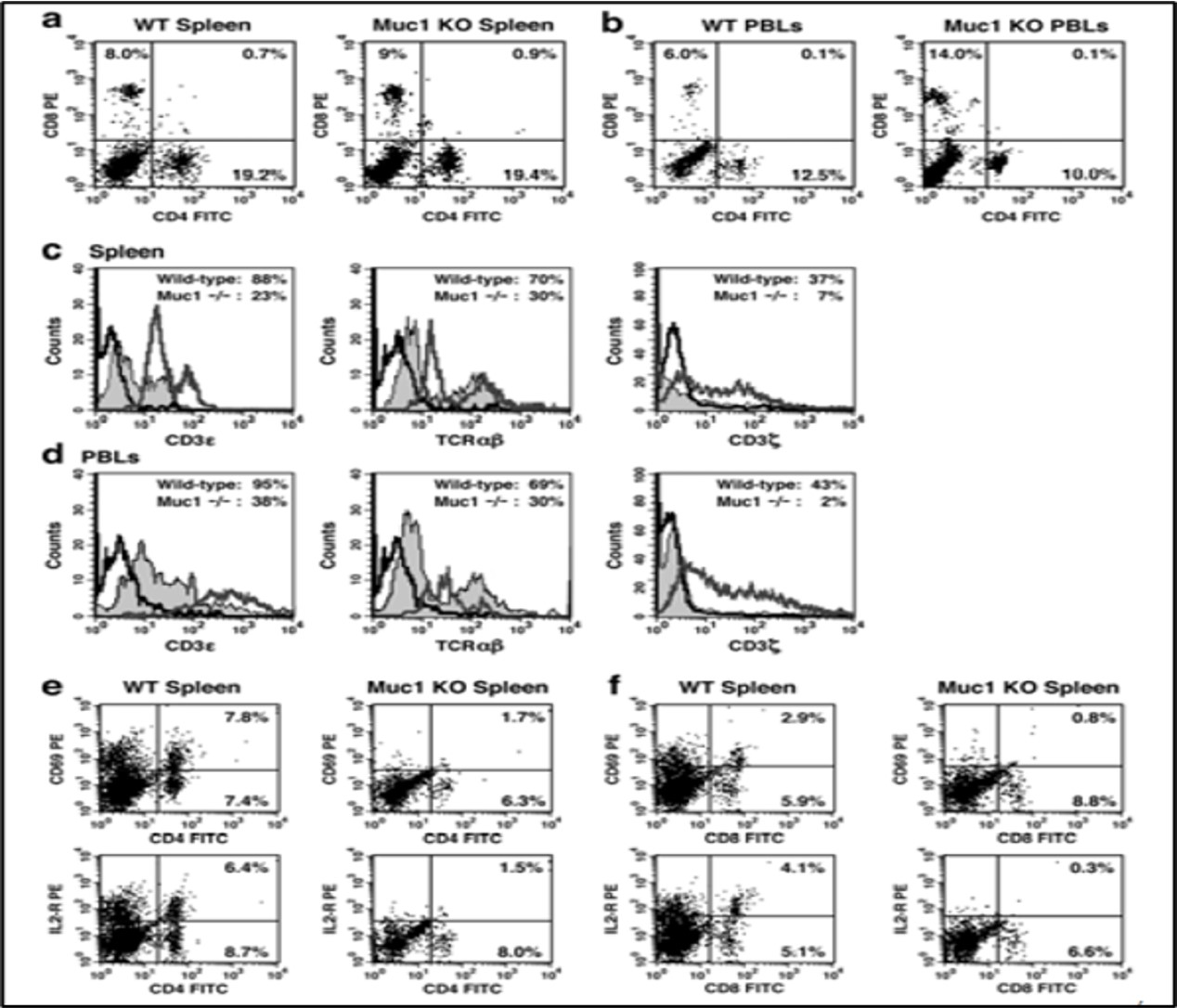

Supplement: Supplementary file 1 [file ijms-22-05587-s001.zip › Supplemental figure 1.jpg]

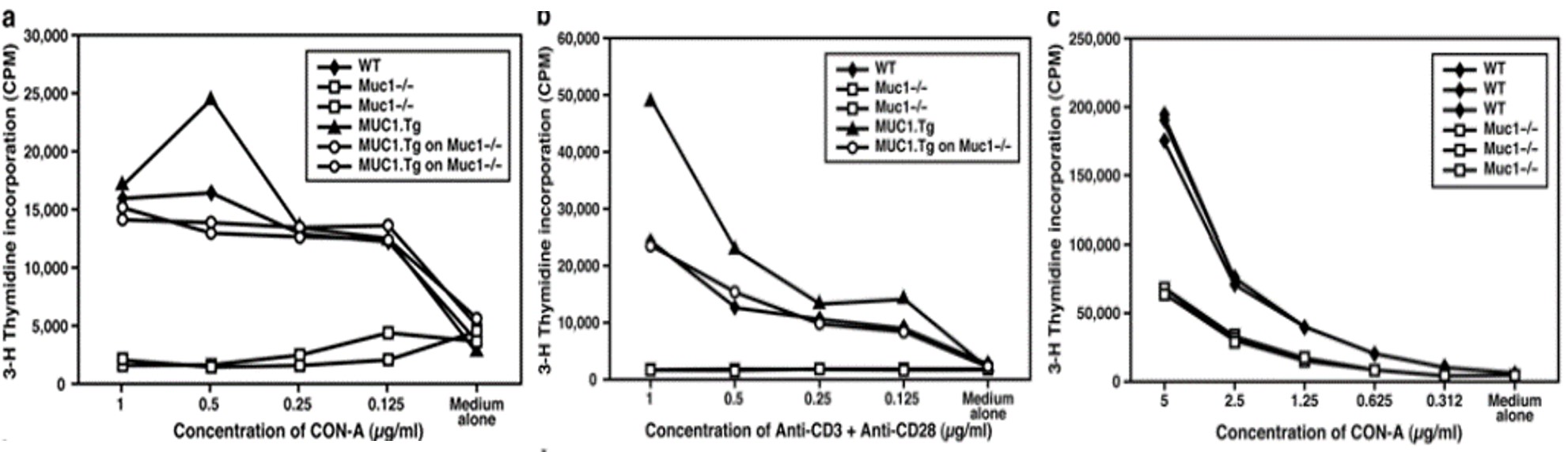

Supplement: Supplementary file 1 [file ijms-22-05587-s001.zip › Supplemental figure 2.jpg]

Significant Pathways in Muc1 vs KO

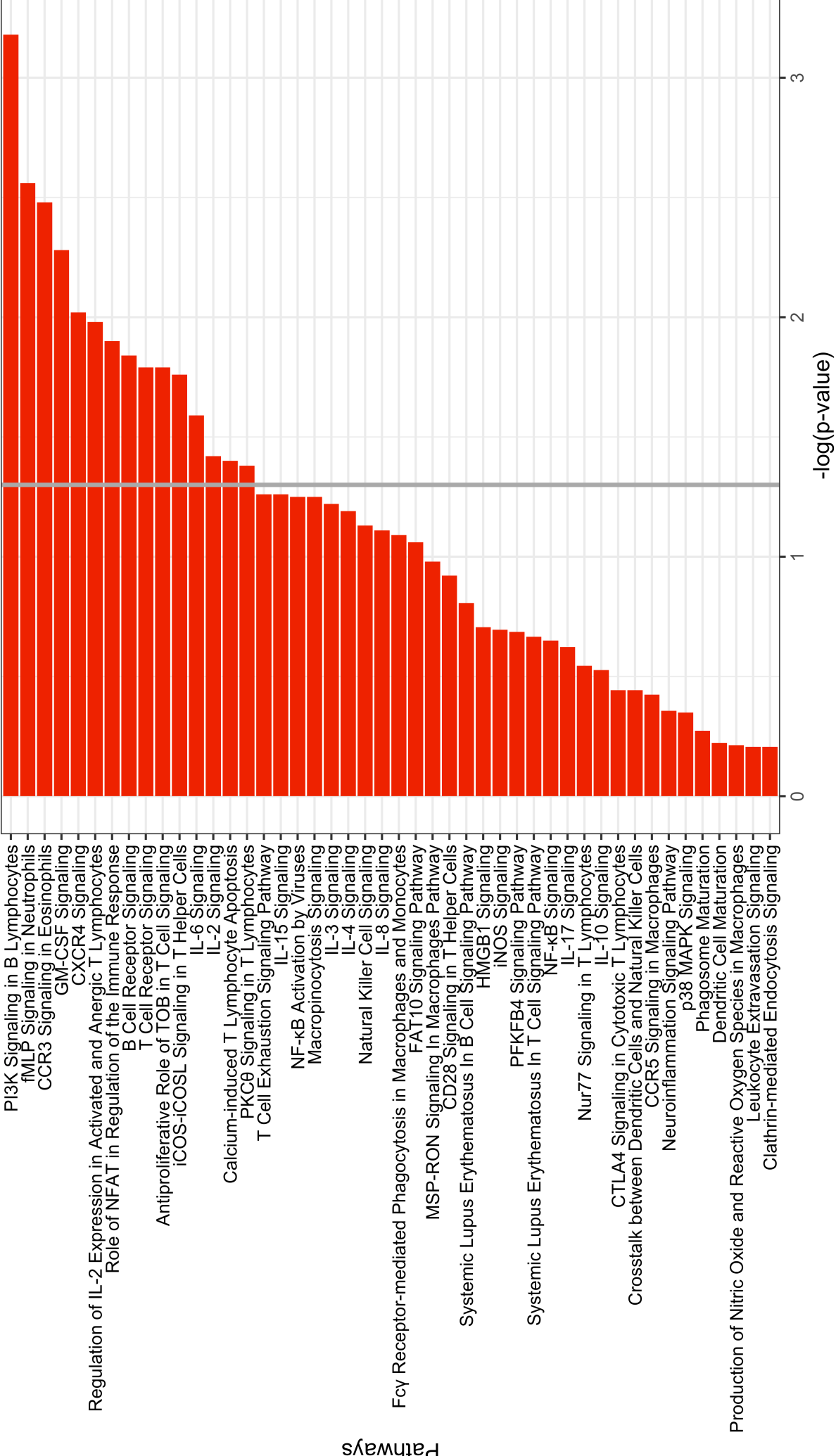

Supplement: Supplementary file 1 [file ijms-22-05587-s001.zip › Supplemental figure 3a.pdf]

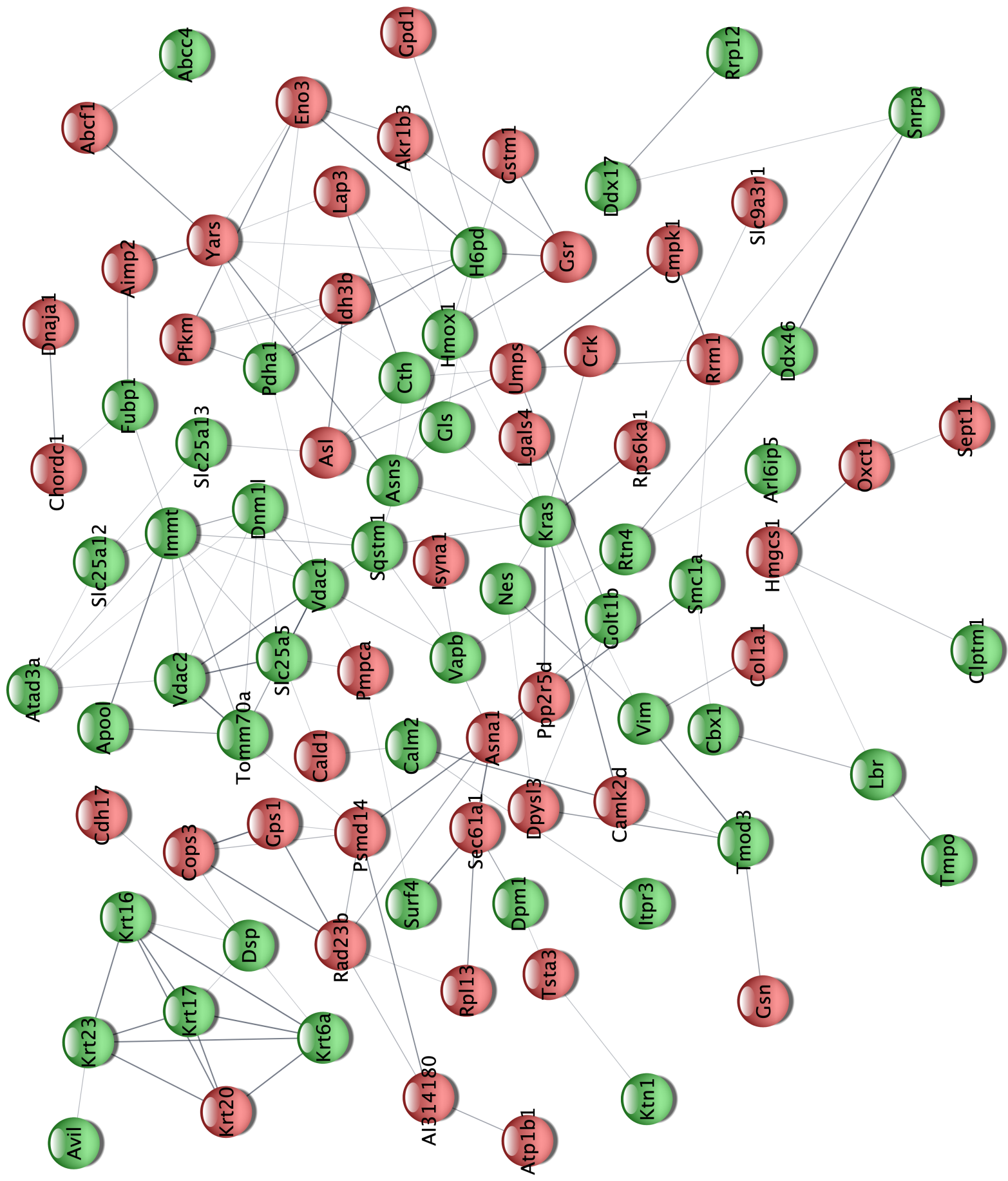

Supplement: Supplementary file 1 [file ijms-22-05587-s001.zip › Supplemental figure 3b.pdf]

## Slide 1
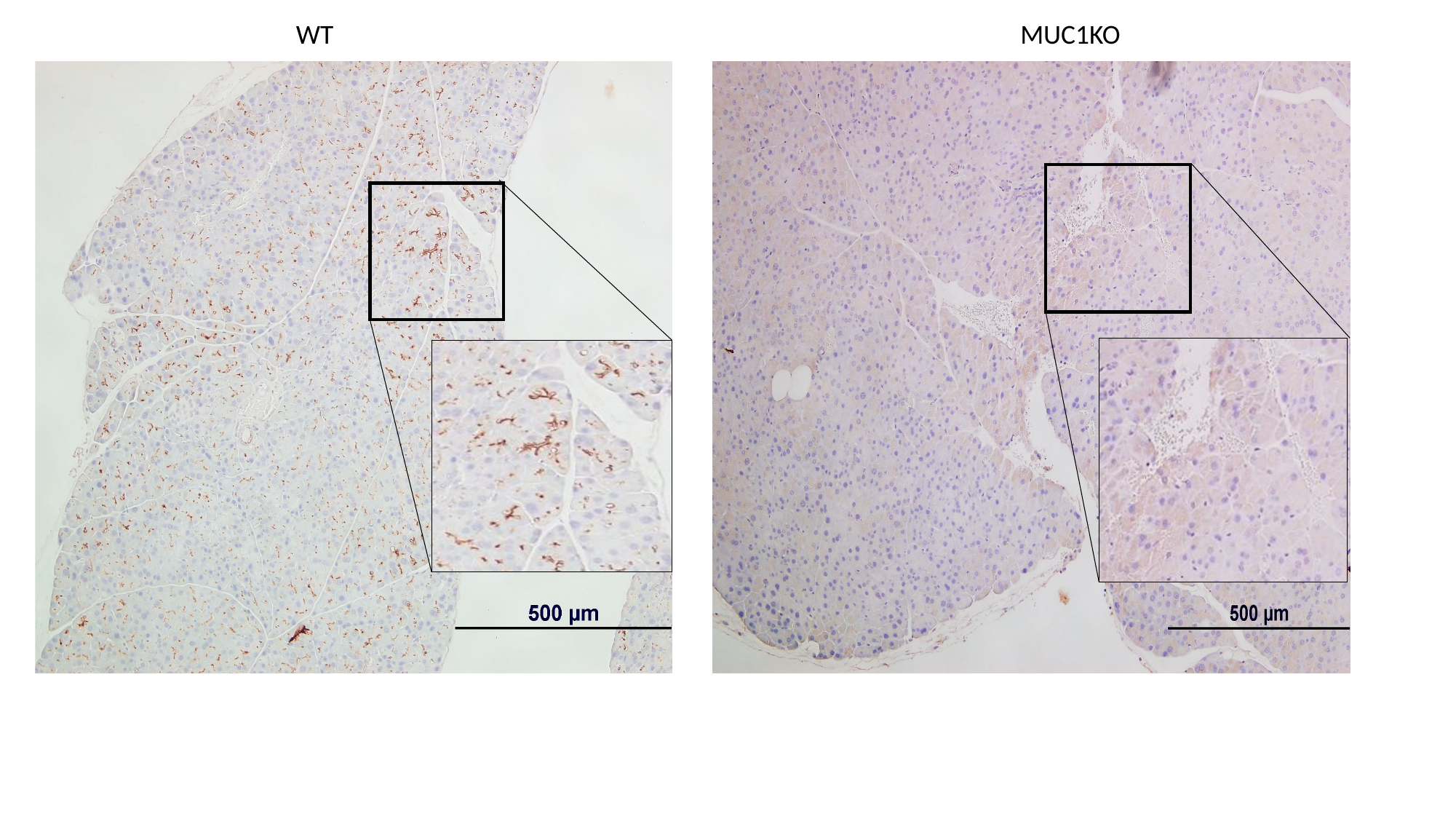

WT
MUC1KO

## Slide 2
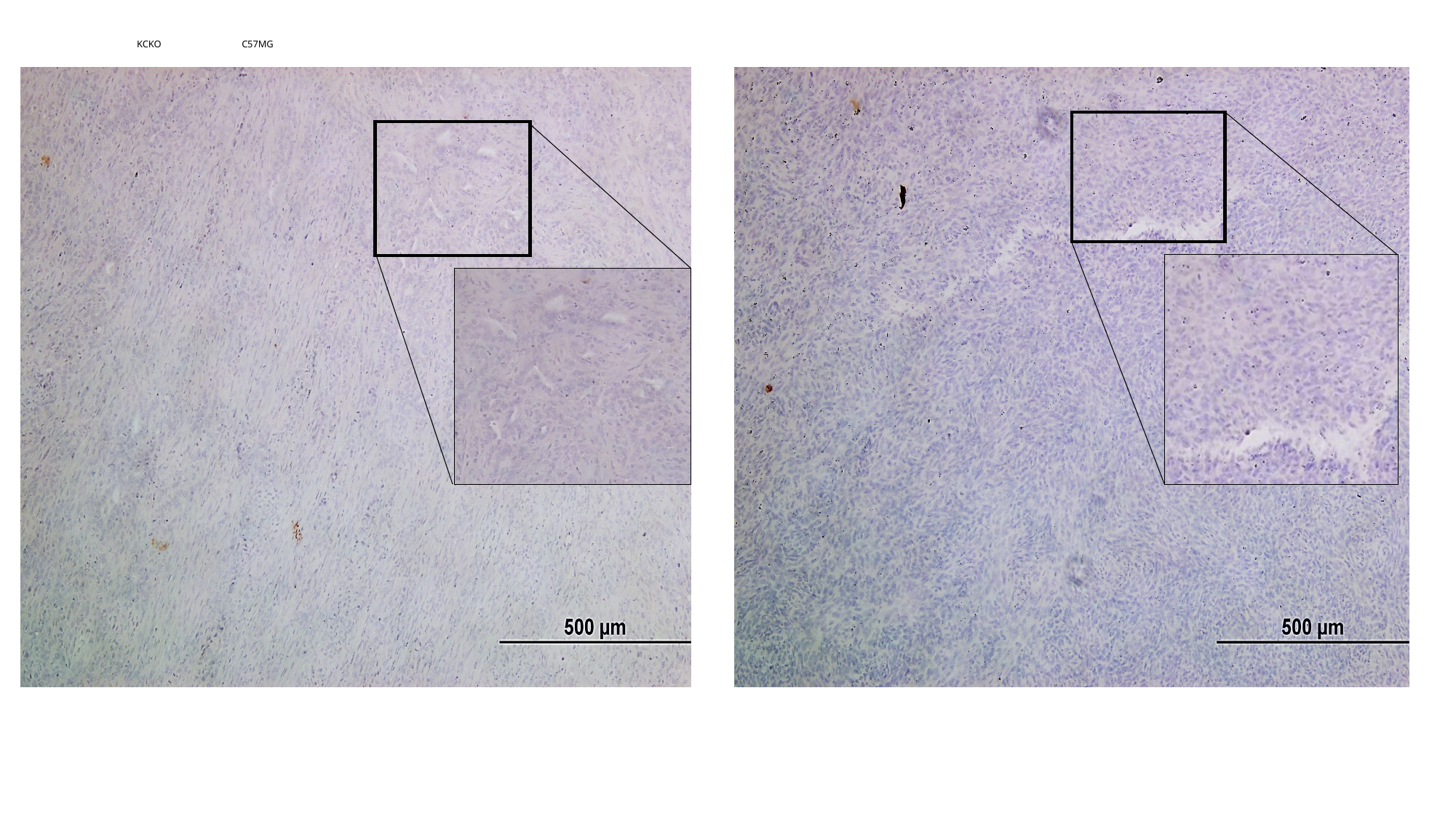

# KCKO C57MG

Supplement: Supplementary file 1 [file ijms-22-05587-s001.zip › Supplemental figure 4.pptx]
